# Ultra-processed food intake and male reproductive function: a mini-review

**DOI:** 10.3389/fnut.2026.1852283

**Published:** 2026-08-27

**Authors:** Jian Huang

**Affiliations:** Clinical Laboratory Center, The First Affiliated Hospital of Guangxi Medical University, Nanning, China

**Keywords:** asthenozoospermia, fecundability, fertility, male reproductive health, reproductive biomarkers, semen quality, subfertility, ultra-processed foods

## Abstract

Ultra-processed foods (UPFs) are a prominent feature of contemporary diets, but their relevance to male reproductive health remains uncertain. This mini-review synthesizes human evidence linking UPF intake with semen quality, asthenozoospermia, fertility-related outcomes, and reproductive biomarkers. Case–control, cross-sectional, prospective, and short-term controlled feeding studies generally report unfavorable associations, most consistently with lower sperm concentration and impaired motility; findings for morphology, semen volume, and other semen domains are less consistent. Prospective evidence links higher paternal UPF intake with lower fecundability and greater subfertility risk, while a controlled feeding trial indicates short-term changes in reproductive hormones, sperm function, and cardiometabolic markers. Interpretation is limited because UPF intake covaries with overall diet quality and lifestyle, exposure metrics and reproductive endpoints are heterogeneous, samples are modest, and clinically meaningful fertility outcomes are scarce. Current findings are therefore suggestive rather than causal. Larger prospective studies with repeated dietary and reproductive assessment, together with longer controlled trials, are needed to determine whether reducing UPF intake as part of broader dietary improvement benefits male reproductive health.

## 1 Introduction

Male reproductive dysfunction has become an important clinical and public health concern worldwide. Infertility affects an estimated 8–12% of couples globally, and male factors are considered a primary or contributing cause in nearly half of cases (1). Population-based evidence further underscores the scale of this burden: the Global Burden of Disease Study estimated 56.5 million prevalent cases of male infertility in 2019, with a global age-standardized prevalence rate of 1402.98 per 100,000, representing a marked increase since 1990 (2). Concern has deepened in recent years because key indicators of male reproductive health appear to be worsening over time. In an updated systematic review and meta-regression of semen samples collected between 1973 and 2018, sperm concentration and total sperm count among unselected men declined by 51.6 and 62.3%, respectively, with evidence that the decline accelerated after 2000 (3).

Against this background, diet has attracted growing attention as a modifiable determinant of male reproductive health (4–11). Evidence on healthy dietary patterns, including Mediterranean-style diets, generally suggests more favorable semen parameters, although it remains largely observational (4–11). The NOVA framework classifies foods according to the nature, extent, and purpose of industrial processing and defines ultra-processed foods (UPFs) as industrial formulations assembled largely from food-derived substances, often with cosmetic additives (12–14). Spermatogenesis and sperm function are sensitive to nutritional, metabolic, and oxidative disturbances (15, 16). However, higher UPF intake may reflect several interrelated features of diet, including less favorable nutritional composition, lower consumption of minimally processed foods, and characteristics of industrial formulation, making it difficult in observational studies to distinguish the effects of food processing itself from those of the broader dietary pattern. Emerging human evidence has linked higher UPF intake with poorer semen quality, greater odds of asthenozoospermia, and less favorable fertility-related outcomes, while short-term intervention data also suggest adverse effects on reproductive and metabolic markers (17–25). However, the evidence remains scattered across heterogeneous study designs and populations, and its overall implications have not been clearly synthesized. This mini-review therefore summarizes the current human evidence on UPF intake and male reproductive function, with a focus on semen quality, asthenozoospermia, fertility-related outcomes, and intervention findings.

## 2 Clinical evidence on ultra-processed food intake and semen quality

### 2.1 Case–control evidence on asthenozoospermia

A 2022 hospital-based case–control study in China specifically examined asthenozoospermia rather than only continuous semen parameters (17). The study included 549 men with asthenozoospermia and 581 controls, and UPF exposure was expressed as the percentage of total energy intake contributed by NOVA-defined ultra-processed foods. Compared with men in the lowest tertile of UPF intake, those in the highest tertile had significantly higher odds of asthenozoospermia, with an adjusted OR of 1.53 (95% CI 1.12–2.10; P for trend <0.05) (17). This association remained directionally consistent in sensitivity analyses using absolute intake rather than energy contribution, suggesting that the finding was not solely an artifact of dietary scaling (17).

Subgroup analyses suggested stronger positive associations among men aged 32 years or older, those with BMI ≥ 24 kg/m^2^, ever smokers, and ever drinkers (17). These findings are compatible with greater vulnerability in the presence of metabolic or lifestyle stressors, but they do not establish effect modification without adequately powered interaction testing (26–31). The clinic-based case–control design also raises selection and reverse-causation concerns. Men aware of fertility problems may have changed their diet, used supplements, or received lifestyle counseling before enrollment; such changes could attenuate, exaggerate, or otherwise distort the observed association (17, 19).

### 2.2 Cross-sectional evidence in healthy and clinic-based men

Subsequent cross-sectional studies largely extended the signal from asthenozoospermia to conventional semen parameters. In the Spanish Led-Fertyl study, 200 healthy men of reproductive age (mean age 28.4 ± 5.5 years) were classified according to the percentage of energy derived from UPFs (18). Men in the highest tertile had lower sperm concentration than those in the lowest tertile and lower total motility; in addition, each 10% increment in energy from UPFs was associated with a reduction in total sperm count (18). A particularly useful feature of this study was the substitution analysis: replacing 10% of energy from UPFs with unprocessed or minimally processed foods was associated with more favorable total sperm count, concentration, total motility, progressive motility, and normal morphology, giving the findings greater dietary and clinical interpretability (18).

The Italian study by Ceretti et al. (20) examined 126 healthy, normal-weight young men. In that analysis, men with the highest UPF intake had markedly lower sperm concentration and lower progressive motility than those with the lowest intake (20). The effect sizes were larger than those reported in Led-Fertyl, which may reflect differences in population structure, exposure metric, or analytical strategy, but the direction of association was clearly concordant (18, 20). Together, these two studies provide cross-sectional support for an inverse association between UPF intake and sperm concentration and motility in otherwise relatively healthy men (18, 20).

More clinically selected populations have yielded a less uniform picture. In a 2025 Iranian cross-sectional study of 260 men recruited from an infertility center, the fully adjusted analysis identified a significant association only for abnormal sperm concentration, and specifically in the second tertile of UPF intake rather than the highest tertile (21). By contrast, no statistically robust associations were observed for motility or morphology deficits (21). This non-monotonic dose–response pattern warrants cautious interpretation, although the finding for sperm concentration was directionally consistent with other studies (21).

Another cross-sectional study conducted in Padova included 358 men aged 18–60 years (mean age, 34.6 ± 9.3 years) who attended a university-based andrology and reproductive medicine unit for semen analysis (22). UPF intake, expressed as the percentage of total energy intake derived from UPFs, was categorized into quartiles. Compared across the lowest and highest quartiles, mean sperm concentration, total sperm count, progressive motility, and the percentage of sperm with typical morphology were significantly lower with higher UPF intake, whereas semen volume and hormonal parameters did not differ significantly across UPF categories (22). In analyses stratified by FSH level, increasing UPF intake was associated with lower sperm concentration, total sperm count, and progressive motility among men with FSH < 8 IU/mL, but not among those with FSH ≥ 8 IU/mL. Typical morphology decreased across UPF quartiles in both FSH strata, whereas findings for sperm viability were less consistent (22).

Not all datasets have replicated these findings. In a 2025 conference abstract from the Environment and Reproductive Health (EARTH) Study, 343 men attending a fertility center contributed 896 semen samples, and median UPF intake was 5.7 servings/day (23). UPF intake was not associated with any of the conventional semen parameters examined, including semen volume, total sperm count, sperm concentration, total or progressive motility, and normal morphology (23). However, the evidentiary weight of this finding is limited because only a conference abstract is available, and the full protocol, exposure assessment and handling, missing-data procedures, sensitivity analyses, and complete results cannot be critically appraised. Until a full peer-reviewed report is available, the finding should be considered preliminary and given less weight than evidence from fully reported studies (19, 23).

### 2.3 Consistency and heterogeneity across semen parameters

Across studies, the most consistent adverse associations concern sperm concentration and total or progressive motility (18, 20–23). Total sperm count generally shows a similar pattern, whereas semen volume shows little consistent association and morphology remains heterogeneous (20–23). Because concentration, count, motility, and morphology are correlated but non-equivalent endpoints, concordant findings should not be treated as independent replications of a single biological effect. Conversely, an isolated association among many tested outcomes warrants caution. The evidence therefore favors a domain-specific interpretation rather than uniform deterioration of semen quality, although the small number of studies does not permit confident ranking of endpoint sensitivity.

## 3 Clinical evidence on fertility outcomes and reproductive biomarkers

### 3.1 Prospective evidence on fecundability and subfertility

A 2026 population-based prospective cohort from the Netherlands examined couple-level fertility outcomes rather than semen parameters alone (25). The analysis included 831 women and 651 male partners; paternal UPF intake was assessed by a validated food frequency questionnaire and expressed as the percentage by weight of total intake contributed by NOVA-defined UPFs (25). Subfertility was defined as time to pregnancy of at least 12 months or conception requiring assisted reproductive technology, and 254 couples (30.6%) met this definition (25). The prospective framework strengthens temporality relative to the case–control and cross-sectional evidence, although dietary assessment still occurred during early pregnancy as a proxy for periconceptional intake (19, 25).

Each one-standard-deviation increase in paternal UPF intake was associated with lower fecundability and higher odds of subfertility in continuous models (25). Associations persisted after mutual adjustment for the female partner’s UPF intake and measured sociodemographic and lifestyle factors, reducing—but not eliminating—the possibility that paternal UPF intake merely represented shared couple-level diet quality (25). Interpretation remains constrained because diet was measured once, only couples who conceived were eligible for analysis, and physical activity was not included in the principal adjustment set; selection, measurement error, and residual confounding therefore remain possible (25). Even with these limitations, this study provides prospective evidence linking paternal UPF intake with clinically meaningful fertility outcomes.

### 3.2 Controlled human evidence on hormonal and functional changes

A 2025 controlled 2 × 2 crossover feeding trial tested whether an ultra-processed dietary pattern could affect male reproductive and metabolic health under standardized conditions (24). Forty-three healthy men aged 20–35 years consumed ultra-processed and unprocessed diets for 3 weeks each, separated by a 12-week washout; half also received 500 kcal/day of excess energy (24). The factorial crossover design helps separate effects associated with the UPF dietary pattern from caloric surplus, but it does not isolate processing alone because the diets also differed in food matrix, ingredients, palatability, and potential food-contact exposures.

Compared with the unprocessed diet, the UPF diet increased body weight and worsened the LDL:HDL ratio independently of caloric load (24). Follicle-stimulating hormone and growth/differentiation factor 15 decreased, while sperm quality showed a non-definitive trend toward impairment, including lower total motility (24). These findings support short-term physiological effects under controlled feeding, but they should not be extrapolated directly to infertility. The trial was small, lasted only 3 weeks per diet, and was not designed to assess time to pregnancy, assisted reproductive outcomes, or sustained changes across a full spermatogenic cycle. Under the trial conditions, these findings provide experimental evidence of short-term changes in selected intermediate outcomes, without identifying the responsible dietary attribute or establishing clinical reproductive harm (24, 32, 33). Table 1 summarizes the key human studies reviewed.

**Table 1:** Summary of key human studies discussed in this review on ultra-processed food intake and male reproductive outcomes.

Study	Country	Design	Population	UPF assessment	Reproductive outcome(s)	Main findings	Key limitations/notes
Lv et al. (17)	China	Hospital-based case–control	549 men with asthenozoospermia and 581 controls	NOVA-defined UPF intake expressed as % of total energy; sensitivity analyses used absolute amount (g/day)	Asthenozoospermia	Highest vs. lowest tertile: adjusted OR 1.53 (95% CI 1.12–2.10); association remained directionally consistent in sensitivity analyses; signal appeared stronger in older, higher-BMI, smoking, and drinking subgroups	Clinic-based sample; residual confounding and selection bias cannot be excluded
Valle-Hita et al. (18)	Spain	Cross-sectional	200 healthy men of reproductive age	UPF intake classified by % energy from UPFs; 10% substitution models also examined	Sperm concentration, total sperm count, total motility, progressive motility, and morphology	Higher UPF intake associated with lower sperm concentration and lower total motility; each 10% increment in UPF energy linked to lower total sperm count; replacing UPFs with unprocessed or minimally processed foods was associated with more favorable semen parameters	Cross-sectional design; small sample size
Ceretti et al. (20)	Italy	Cross-sectional	126 healthy, normal-weight young men	Higher vs. lower UPF intake categories	Sperm concentration and progressive motility	Highest UPF intake associated with markedly lower sperm concentration and lower progressive motility	Cross-sectional design; small sample size
Soltani et al. (21)	Iran	Cross-sectional	260 men recruited from an infertility center	UPF intake tertiles	Total sperm motility, sperm concentration, sperm volume, and normal sperm morphology	Significant association observed only for abnormal sperm concentration, mainly in the second tertile; no statistically robust association for motility or morphology deficits	Irregular dose–response pattern; clinic-based sample
Petre et al. (22)	Italy	Cross-sectional	358 men aged 18–60 years	UPF intake quartiles	Sperm concentration, total sperm count, progressive motility, morphology, and hormonal parameters	Mean sperm concentration, total sperm count, progressive motility, and typical morphology declined across increasing UPF categories; no significant overall variation in hormonal parameters; FSH-stratified associations differed across semen domains	Cross-sectional design; clinic-based sample
Chavarro et al. (23)	USA	Clinic-based cross-sectional analysis with repeated semen measurements	343 men; 896 semen samples	131-item food frequency questionnaire; NOVA classification; UPF consumption analyzed in quartiles	Semen volume, total sperm count, sperm concentration, total motility, progressive motility, and normal morphology	No clear association between UPF consumption and conventional semen parameters	Congress abstract only; full methods and sensitivity analyses unavailable; evidentiary weight limited
Preston et al. (24)	Denmark	Controlled 2 × 2 crossover feeding trial	43 healthy men aged 20–35 years	Controlled dual-arm 2 × 2 crossover feeding trial: participants assigned to either an adequate-calorie or excess-calorie arm (+500 kcal/day) received 3-week ultra-processed and unprocessed diets in randomized crossover order, separated by a 12-week washout; diets were calorie-matched within each arm.	Reproductive and related metabolic outcomes	The UPF diet increased body weight in both calorie arms and increased the LDL: HDL ratio in the adequate-calorie arm. In the excess-calorie arm, FSH and GDF-15 decreased, while total sperm motility showed a non-significant downward trend; sperm concentration and morphology were not significantly altered.	Small sample and short intervention; not designed to assess clinical fertility outcomes or sustained effects across a full spermatogenic cycle
Lin et al. (25)	Netherlands	Population-based prospective cohort	831 women and 651 male partners	Validated FFQ; UPF intake was expressed as a percentage of total food intake (grams per day)	Fecundability, subfertility (time to pregnancy ≥ 12 months or use of assisted reproductive technology)	Each 1-SD increase in paternal UPF intake was associated with lower fecundability and higher odds of subfertility; associations persisted after mutual adjustment for female partner UPF intake and shared lifestyle factors	Diet measured once in early pregnancy as a proxy for periconceptional intake; only couples who conceived were included; residual confounding remains possible

BMI, body mass index; FFQ, food frequency questionnaire; FSH, follicle-stimulating hormone; GDF-15, growth/differentiation factor 15; LDL: HDL, low-density lipoprotein to high-density lipoprotein ratio; NOVA, food-processing classification system; UPF, ultra-processed food; CI, confidence interval; OR, odds ratio; SD, standard deviation.

## 4 Potential explanatory frameworks

### 4.1 Nutritional composition and dietary displacement

UPF intake is embedded within the broader dietary pattern in which it occurs. Higher intake is generally associated with a less favorable nutritional profile, including higher intakes of added sugars, sodium, and saturated fat and lower intakes of fiber and several micronutrients, as well as lower consumption of unprocessed or minimally processed foods (14, 34–40). Conversely, Mediterranean-style and other healthy dietary patterns—rich in fruits, vegetables, legumes, whole grains, nuts, fish, and unsaturated fats—have been associated with more favorable sperm concentration, total sperm count, and motility, with less consistent findings for morphology (4–11). In the Led-Fertyl study, theoretical replacement of 10% of energy from UPFs with an equivalent energy contribution from unprocessed or minimally processed foods was associated with more favorable semen parameters (18). The Padova study assessed Mediterranean-diet adherence and UPF intake concurrently in the same participants (22). These measures capture related but distinct dimensions of diet: lower UPF intake does not necessarily imply greater adherence to a healthy dietary pattern, and vice versa. Analyses incorporating both measures may help determine whether associations with UPF intake persist after accounting for overall diet quality. These findings are compatible with a dietary-displacement framework but cannot distinguish the contributions of processing-related attributes, nutritional composition, and lower consumption of potentially protective foods. The controlled trial compared calorie-matched UPF and unprocessed diets within both adequate- and excess-calorie arms, but the diets also differed in food-group, ingredient, and nutrient composition and in potential contaminant exposures (24).

### 4.2 Metabolic and inflammatory pathways

Metabolic dysfunction is one possible explanatory pathway. In broader epidemiological literature, higher UPF intake is associated with obesity, dyslipidemia, insulin resistance, and low-grade systemic inflammation (32–34, 36–40), while the crossover trial documented short-term weight gain and deterioration in the LDL:HDL ratio (24). Adiposity, oxidative stress, and inflammatory dysregulation have each been associated with adverse male reproductive outcomes (26–31). However, current UPF studies have not demonstrated mediation through these factors. Depending on their temporal relation to UPF intake and the assumed causal structure, BMI and metabolic factors may function as confounders, mediators, or both; differences in whether and how studies adjust for these variables may therefore contribute to heterogeneity in effect estimates. Subgroup associations observed among older men, men with higher BMI, ever smokers, and ever drinkers should be considered hypothesis-generating; in the absence of adequately powered interaction tests, they do not establish effect modification or identify a distinct susceptible subgroup (17).

### 4.3 Non-nutritional components and food-related exposures

**Figure 1:**
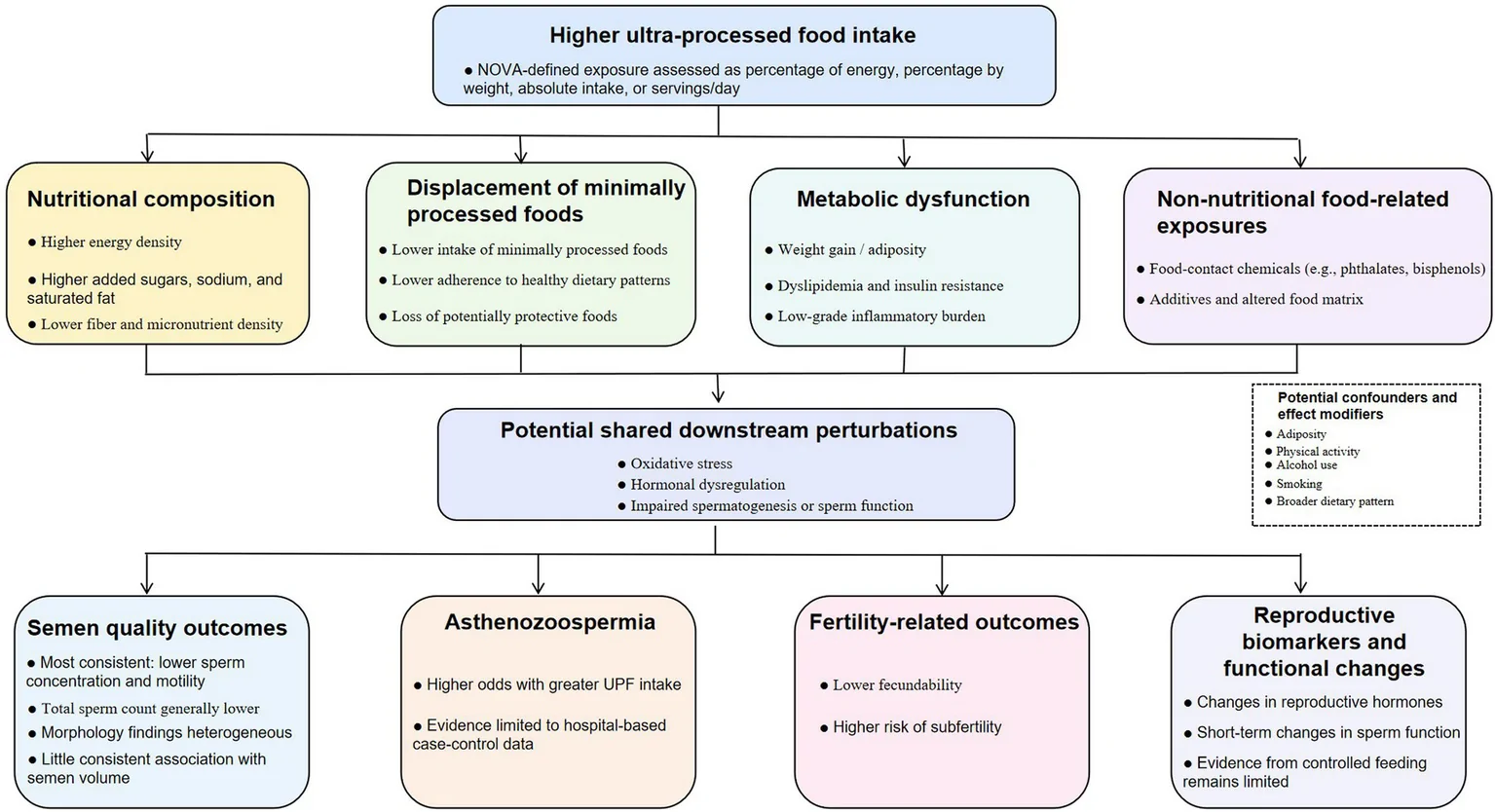
Proposed pathways linking higher ultra-processed food intake to impaired male reproductive function. Ultra-processed food exposure has been assessed using NOVA-based metrics, including percentage of energy intake, percentage by weight, absolute intake, and servings per day. Higher intake may reflect adverse nutritional composition, displacement of minimally processed foods, metabolic dysfunction, and non-nutritional food-related exposures. These overlapping pathways may converge on potential downstream perturbations, including oxidative stress, hormonal dysregulation, and impaired spermatogenesis or sperm function, and may contribute to poorer semen quality, higher odds of asthenozoospermia, lower fecundability, greater subfertility risk, and short-term changes in reproductive biomarkers and sperm function. Adiposity, physical activity, smoking, alcohol use, the broader dietary pattern, and non-dietary sources of chemical exposure may confound or modify the observed associations. Arrows indicate hypothesized relationships rather than independent or established causal pathways.

Potential non-nutritional pathways also warrant consideration, although evidence directly linking them to UPF-related male reproductive outcomes remains limited. In NHANES 2013–2014, higher UPF intake was associated with higher urinary concentrations of several phthalate metabolites, while the controlled feeding trial reported a trend toward higher serum cxMINP after the UPF diet (24, 41). In parallel, the broader male reproductive literature has linked endocrine-disrupting chemicals to hormonal disturbances and adverse semen parameters (42–44). However, these strands of evidence remain indirect and do not demonstrate mediation, because none of the reviewed studies simultaneously assessed UPF intake, chemical biomarkers, and reproductive outcomes, and relevant exposures may also originate from non-dietary sources. Other processing-related features, including additives, altered food matrices, and processing-induced biological responses, have likewise been proposed, but their contribution to human male reproductive outcomes has not been established (12–14, 39, 40). Figure 1 therefore presents these pathways as hypotheses rather than confirmed causal mechanisms.

## 5 Discussion

### 5.1 Overall patterns of association

Three features characterize the current evidence. First, associations are most consistent for sperm concentration and motility, whereas findings for semen volume and morphology are less consistent. Second, broadly similar associations have been reported in both healthy and clinic-based samples, although effect estimates and dose–response patterns vary across studies. Third, prospective and controlled evidence extends beyond conventional semen parameters to fecundability, reproductive hormones, and sperm function, yet neither study establishes that industrial processing itself causes infertility. The available evidence therefore warrants further investigation but does not support causal or therapeutic conclusions.

### 5.2 Methodological heterogeneity and limitations

Interpretation is constrained not only by the predominance of observational designs but also by substantial methodological heterogeneity. Studies include healthy men and patients recruited from infertility clinics; in the latter, underlying diagnoses, treatment-seeking behavior, and pre-enrollment dietary changes may limit generalizability and increase susceptibility to selection bias and reverse causation (17–23). UPF intake has been quantified as percentage of energy, percentage by weight, absolute intake, or servings and categorized using tertiles or quartiles, with exposure assessed by different questionnaires or dietary recalls; these approaches capture different dimensions of diet and yield non-equivalent contrasts (12–14, 17, 18, 20–25). Weight- and energy-based metrics may also rank water-rich and energy-dense products differently. Classification is further complicated by composite dishes and brand-specific products whose formulations may be incompletely captured by dietary instruments. Nondifferential misclassification would generally attenuate associations, whereas differential reporting could bias estimates in either direction. Studies also differ in the number of semen samples collected, control of abstinence duration, laboratory procedures, handling of repeated measurements, and the WHO manual applied. Standardized WHO procedures are intended to improve interlaboratory comparability (45), yet repeated analyses in 5240 male partners of subfertile couples showed within-person coefficients of variation of 28–34% across semen parameters (46). A single sample may therefore poorly characterize an individual’s usual semen profile, whereas repeated samples require methods that account for within-person clustering. Covariate sets vary in whether they include total energy intake, physical activity, BMI, metabolic factors, overall diet quality, and other NOVA groups. Such differences may leave residual confounding, whereas adjustment for variables on the causal pathway may attenuate the total effect of UPF intake. Moreover, most estimates represent relative dietary contrasts without specifying which foods replace UPFs; without substitution analyses, they do not correspond directly to a feasible dietary intervention. Regional context also matters because foods classified as UPFs, their nutritional composition, and potential food-contact exposures differ across China, Mediterranean Europe, Iran, the Netherlands, and the United States. Finally, most studies are modest in size, assess multiple correlated semen outcomes, and rarely use repeated dietary measurements or adjust for multiple comparisons. Given these differences, quantitative pooling of the current evidence may yield an estimate of uncertain interpretation; qualitative agreement should not be mistaken for equivalence of effect estimates (17–25).

### 5.3 Clinical and public health implications

The evidence has potential clinical relevance but does not justify recommending UPF restriction as a stand-alone treatment for male infertility (19, 47–49). Dietary counseling may reasonably emphasize replacing frequently consumed UPFs with unprocessed or minimally processed foods within an overall healthy dietary pattern, together with weight management, physical activity, smoking cessation, and moderation of alcohol intake (4–6, 8–11, 26, 27, 30, 31, 47–49). This approach avoids attributing all potential benefit to processing, acknowledges nutritional heterogeneity within NOVA categories, and complements rather than replaces standard infertility evaluation and treatment. Patients should also be informed that direct evidence that reducing paternal UPF intake improves pregnancy or live-birth outcomes is not yet available.

### 5.4 Priorities for future research

Future studies should use prospective preconception designs with repeated assessments of diet and semen, standardized abstinence and laboratory procedures, and clinical outcomes including time to pregnancy, miscarriage, assisted reproduction, pregnancy, and live birth (19, 25, 47–49). Investigators should prespecify causal models that distinguish confounders from potential mediators and, where appropriate, report total-effect estimates alongside models accounting for adiposity and metabolic factors. Analyses should compare energy-, weight-, and serving-based UPF metrics, account for overall dietary patterns and physical activity, and evaluate substitutions with unprocessed or minimally processed foods. Characterizing UPF subgroups and regional product composition may clarify whether associations are driven by particular foods rather than the category as a whole (12–14, 39, 40). Longer controlled feeding trials spanning a substantial proportion of the spermatogenic cycle are needed to assess persistence and reversibility and to examine potential metabolic, hormonal, oxidative, and food-contact pathways (19, 24, 32). Where feasible, preregistration, harmonized reporting, and publication of null findings could reduce selective reporting and improve comparability across studies.

## 6 Conclusion

Current human evidence suggests that higher UPF intake is most consistently associated with lower sperm concentration and motility, while limited prospective evidence also suggests lower fecundability and greater subfertility risk. However, the evidence base remains small, heterogeneous, and largely observational and cannot disentangle the effects of industrial processing from those of nutritional composition, lower consumption of minimally processed foods, and correlated lifestyle factors. Reducing UPF intake should therefore be framed as part of broader dietary improvement rather than as a specific treatment for male infertility. Larger prospective studies with repeated dietary and reproductive assessments, together with longer controlled feeding trials, are needed to determine whether these associations are causal and reversible and whether reducing UPF intake improves pregnancy and live-birth outcomes.
